# Intelligent health system for investigation and consenting COVID-19 patients and precision medicine

**DOI:** 10.2217/pme-2021-0068

**Published:** 2021-10-08

**Authors:** Zeeshan Ahmed

**Affiliations:** 1 ^1^Rutgers Institute for Health, Health Care Policy & Aging Research, Rutgers University, 112 Paterson Street, New Brunswick, NJ 08901, USA; 2 ^2^Department of Medicine, Robert Wood Johnson Medical School, Rutgers Biomedical & Health Sciences, 125 Paterson Street, New Brunswick, NJ 08901, USA

**Keywords:** artificial intelligence, COVID-19, data analytics, machine learning, patients recruitment

## Abstract

Advancing frontiers of clinical research, we discuss the need for intelligent health systems to support a deeper investigation of COVID-19. We hypothesize that the convergence of the healthcare data and staggering developments in artificial intelligence have the potential to elevate the recovery process with diagnostic and predictive analysis to identify major causes of mortality, modifiable risk factors and actionable information that supports the early detection and prevention of COVID-19. However, current constraints include the recruitment of COVID-19 patients for research; translational integration of electronic health records and diversified public datasets; and the development of artificial intelligence systems for data-intensive computational modeling to assist clinical decision making. We propose a novel nexus of machine learning algorithms to examine COVID-19 data granularity from population studies to subgroups stratification and ensure best modeling strategies within the data continuum.

The quest to comprehend COVID-19 is the central focus of humankind today [1]. We have now realized that to effectively diagnose and treat patients with COVID-19 symptoms, we need an evolving understanding of the complex nature and course of the disease. [2,3]. Healthcare data analysis has the potential to transform the healthcare sector by predicting vulnerabilities and tailoring COVID-19 prevention and treatment [4]. In the past decades, various systems have been developed in academic and commercial sectors, but both are unable to identify problems by their effects and significantly help in clinical decision making with the healthcare data analytics and timely academic research collaboration [5–7]. Our central hypothesis states that integrative, intelligent and analytic access to the healthcare data of high volume, velocity, variety and veracity has the potential to revolutionize the field of medicine by providing the best strategies to diagnose and treat COVID-19 patients, especially those at risk of serious medical complications arising from a better understanding of the biology [8]. However, current limitations imply a gap in clinical and academic settings, difficulties in getting exigent approvals and timeliness of data availability, levels of granularity in clinical information and application of appropriate modeling strategies that allow learning in the data continuum. To efficiently establish a COVID-19 investigation, we need to deal with the major barriers to successfully implementing artificial intelligence (AI) in the healthcare sector, which include devising patient-centric protocols to improve the enrolment rates; translational integration of electronic health records (EHR) and diversified publicly available datasets with optimization tools [9]; and the development of a Health Insurance Portability and Accountability Act-compliant AI system for data-intensive computational modeling to assist clinical decision making [10].

We need innovative studies supporting the development of multifunctional machine learning frameworks to address complex and big data-oriented challenges in the healthcare sector by categorizing interaction patterns among variables, learning from experiences and predicting better orientations, which can support clinicians by efficiently stratifying subjects to understand specific scenarios related to COVID-19 and optimize decision making [11]. It will support constructing cohorts and ontologies from COVID-19 patient population based on personal, regional and clinical information. Here, another challenge is to bridge clinical practices and academic research environments with the development of an efficient, secure and effective patient’s recruitment program. We need advanced frameworks to support consenting COVID-19 patients with the collection of clinical phenotypic data for the establishment of emerging and collaborative COVID-19 studies. A specialized data management system is required to facilitate and improve overall COVID-19 patient recruitment, consenting and collection and tracking of original and current quantities of biospecimens. We must foster cross-disciplinary collaborative research environments that will enable clinicians and researchers to gain a complete picture of the COVID-19 patient to deepen their understanding and positively impact treatment decisions [1]. We propose implementing cutting-edge technologies utilizing AI approaches for multimodal data aggregation, classification and the development of a knowledge base of clinical predictors for decision support (Figure 1). We need to design and develop intelligent systems that support COVID-19 patient recruitment, consent for research and relevant healthcare data management and analysis including features to build prospective cohorts and ontologies from the COVID-19 population data.

**Figure 1.**
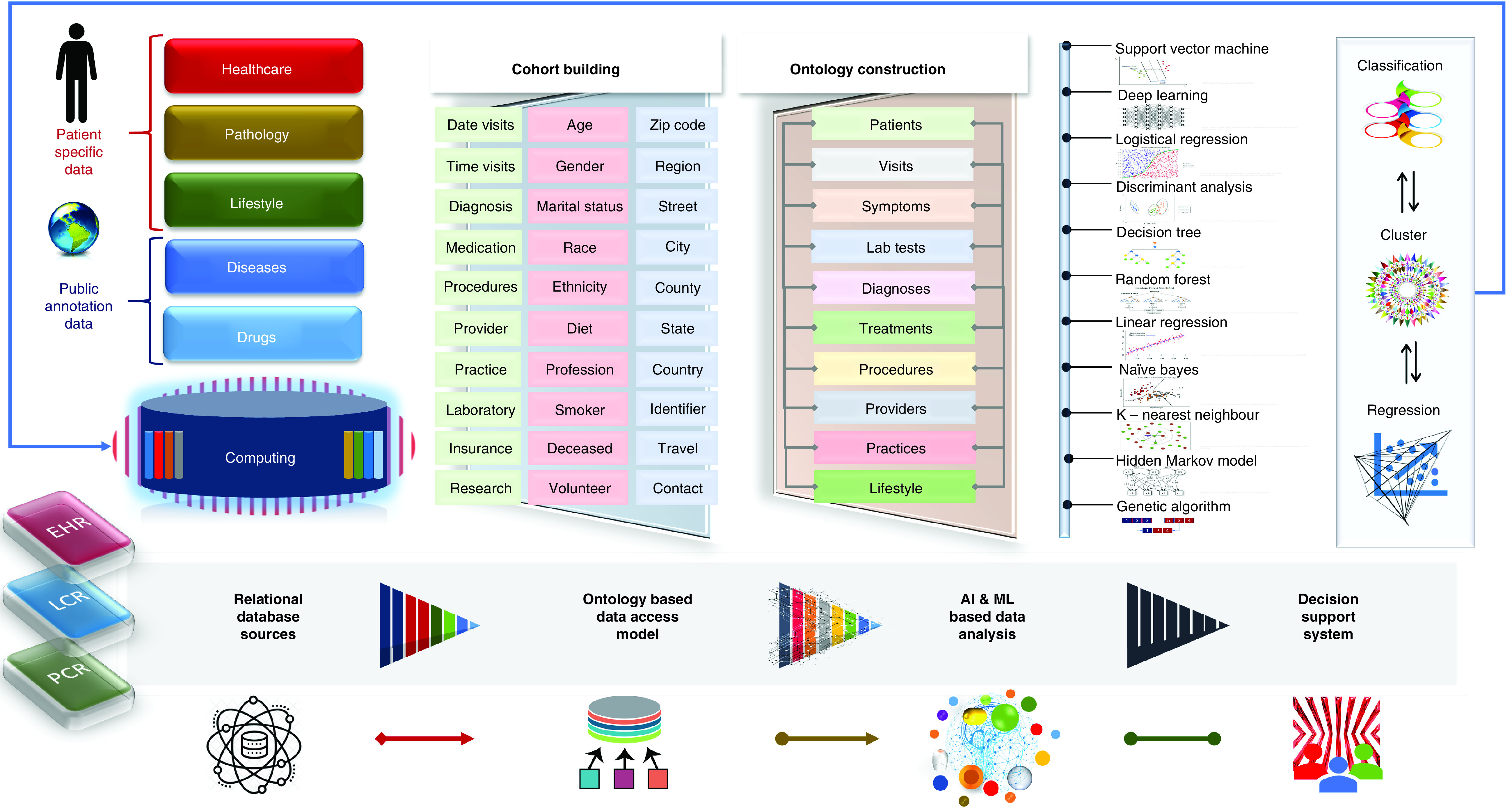
Research methodology. **(A)** Relational database modeling within computing environment for patient-specific and publicly available annotated data management. **(B)** Cohort building and ontology-based data access modeling. **(C)** Intelligent data analysis with AI and machine learning approaches; and **(D)** decision support system development. AI: Artificial intelligence; EHR: Electronic health records; LCR: Lifetime clinical records; ML: Machine learning; PCR: Polymerase chain reaction.

## COVID-19 patient recruitment for research

To efficiently establish COVID-19 investigation, it is important to design patient-centric protocols to improve the recruitment and retention process. Transparent and timely access to data and sample collection from patients is central to combat against epidemics and pandemics [12]. With most of the currently deployed processes in clinics, it is very difficult for the investigators in academia to get information about COVID-19 patients visiting practices and out of those who are willing to volunteer to participate in research and clinical trials [13]. It is highly required to have a system that can bridge this gap and facilitate the establishment of new COVID-19 studies [14].

We need effective specialized data management systems to improve and accelerate overall COVID-19 patient recruitment, consent and integrated healthcare data analytics processes. It would be health measurement platforms to efficiently support analyzing a wide range of patient-consented clinical data to identify qualified patients for COVID-19 studies. It should be able to support handling of clinical phenotypic data (e.g., health information from longitudinal medical records) and specimen materials (e.g., plasma, serum, nasopharyngeal swabs, tissue, blood, etc.) and their derivatives (e.g., DNA, RNA, stem cells, etc.) needed for the development and application of emerging technologies to study problems in COVID-19. Such systems will help in developing markers for diagnosis, prognosis and prediction of response to therapy, as well as provide a critical resource to facilitate translational research. These resources should be open source and will likely to be used by numerous collaborating groups within the medical and research communities, especially those for whom the development of translational COVID-19 research projects has become a major goal.

**Figure 2.**
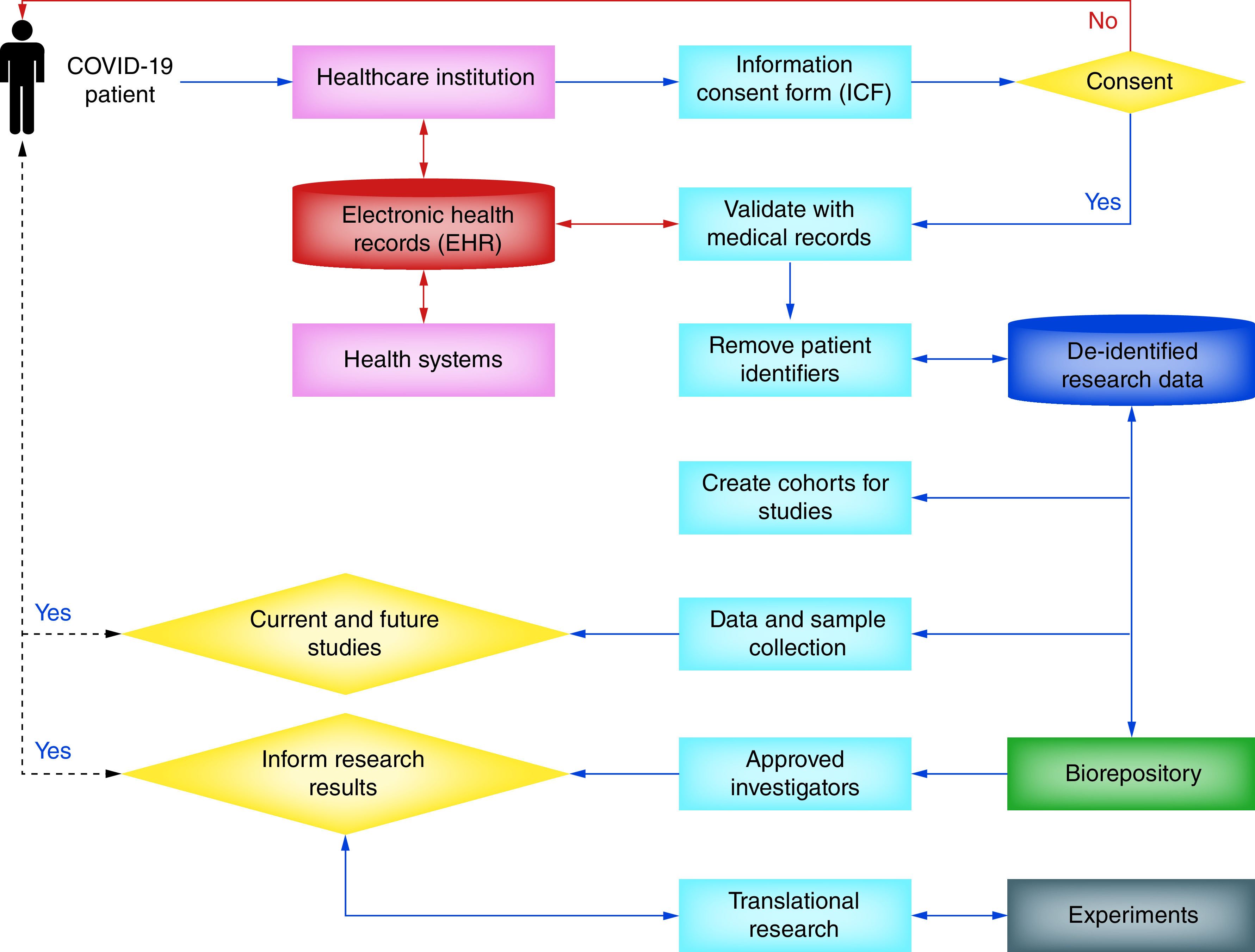
COVID-19 patient consent process for research.

To support healthcare and computational communities, we propose a workflow (Figure 2) that starts with an electronic and paper-based information consenting form (ICF), to be completed by the COVID-19 patients at their visits to the practices, with or without help of staff assisting. It can be developed as a biomedical informatics application (e.g., desktop, mobile, web), which COVID-19 patients can use to start or end their participation in the relevant research study. Overall conceptual design is based on different steps, which starts with patients who are interested in joining having to sign a copy of the ICF, before their information and sample gets collected for research. It supports screening COVID-19 patients using defined criteria, which may include patients of at least 18 years of age. Next, it requests participants to provide their permission for the use of their provided information for retrospective studies and for the principal investigator, the honest broker and the biorepository staff to review this information to determine whether the participants are eligible for future studies. Our approach asks participants for their permission to allow research staff to contact them to ascertain their interest in participating in future studies if deemed eligible. Furthermore, it asks whether participants would like to be informed of the results of retrospective COVID-19 research studies involving the use of their medical record information, including information contained (if any) within the biorepository.

Once the consent is obtained and approved by a staff member (e.g., staff, nurse etc.), copies of the consent forms will be maintained in the COVID-19 patient medical records with restricted access and appropriate security. Potential risks of participation can be addressed, which may include, but are not limited to, secure unique identifiers, which will be secured with restricted access. It supports removing direct participant identifiers (i.e., names, social security numbers, medical record numbers etc.) from information stored with replacements assigned. At no time will any identifiable information be provided to any researcher. However, COVID-19 patients may consent to be contacted by the healthcare institution. If the COVID-19 patient agrees to be informed, a staff member may contact the patient in the future to see if they would be willing to participate in an additional study. The COVID-19 patient will be given the information to contact the researchers directly. De-identified data of the patients who will participate in any kind of basic/life science COVID-19 studies in collaboration with any molecular and pathology labs, can be made available for experimentations and integrative analysis.

We recommend modeling databases of clinical and laboratory and related data linking to the ICF and collected samples to facilitate future research studies and pilot analyses related to COVID-19 research. It will support the development of a secure and friendly graphical interface for users (administrator, staff, nurses, physicians and investigators etc.) and COVID-19 patients (including approved caregivers). Those COVID-19 patients, who need to be monitored while participating in any study, can be provided access to the further survey forms and automated response recording mechanisms (e.g., monitoring pulse, sleep, walking, distance to the practice etc.). Depending upon medical and non medical needs, it will provide universal assessment among COVID-19 patient groups for clinical services that re-interested. All information gathered while recruitment, consenting and monitoring processes can be linked to the medical records (e.g., EHR database or any other medical and claims database). This will facilitate COVID-19 patient verification and pulling of medical details of patients consented for research, data which can be further stratified based on race, ethnicity, gender, age, diagnoses, symptoms, treatments etc. Our proposed model can support building requested cohorts by the approved investigators to establish research studies. It promotes sending invites to the COVID-19 patients from the cohort (any/all) for volunteer participations by pulling their contact information from the medical records (if possible), which may include mailing address, email, telephone and mobile numbers. It can also assist Institutional Review Board process by timely identifying information of COVID-19 patients agreed for participating in any study that will be conducted at associated labs and institutions.

## Intelligent healthcare data analysis

With current progress, AI is still at a preliminary stage and has not yet been impactful against COVID-19. Reasons include inefficient handling of scattered, unstructured, noisy and outlier clinical data; inappropriate selection and application of AI approaches; distorted patient recruitment and consenting process; and governance of data privacy concerns and public health imperatives [5]. Still, we envision utilizing the potential of AI and machine learning (ML), which can help diagnoses and predict the spread of the COVID-19 infection, search for the best treatments among available, reduce medical cost, guide admissions and discharge and social control [1,6]. However, we need to examine how COVID-19 data granularity relates to multiple AI approaches and reconcile the interoperability and learning to ensure best modeling strategies within the data continuum.

**Figure 3.**
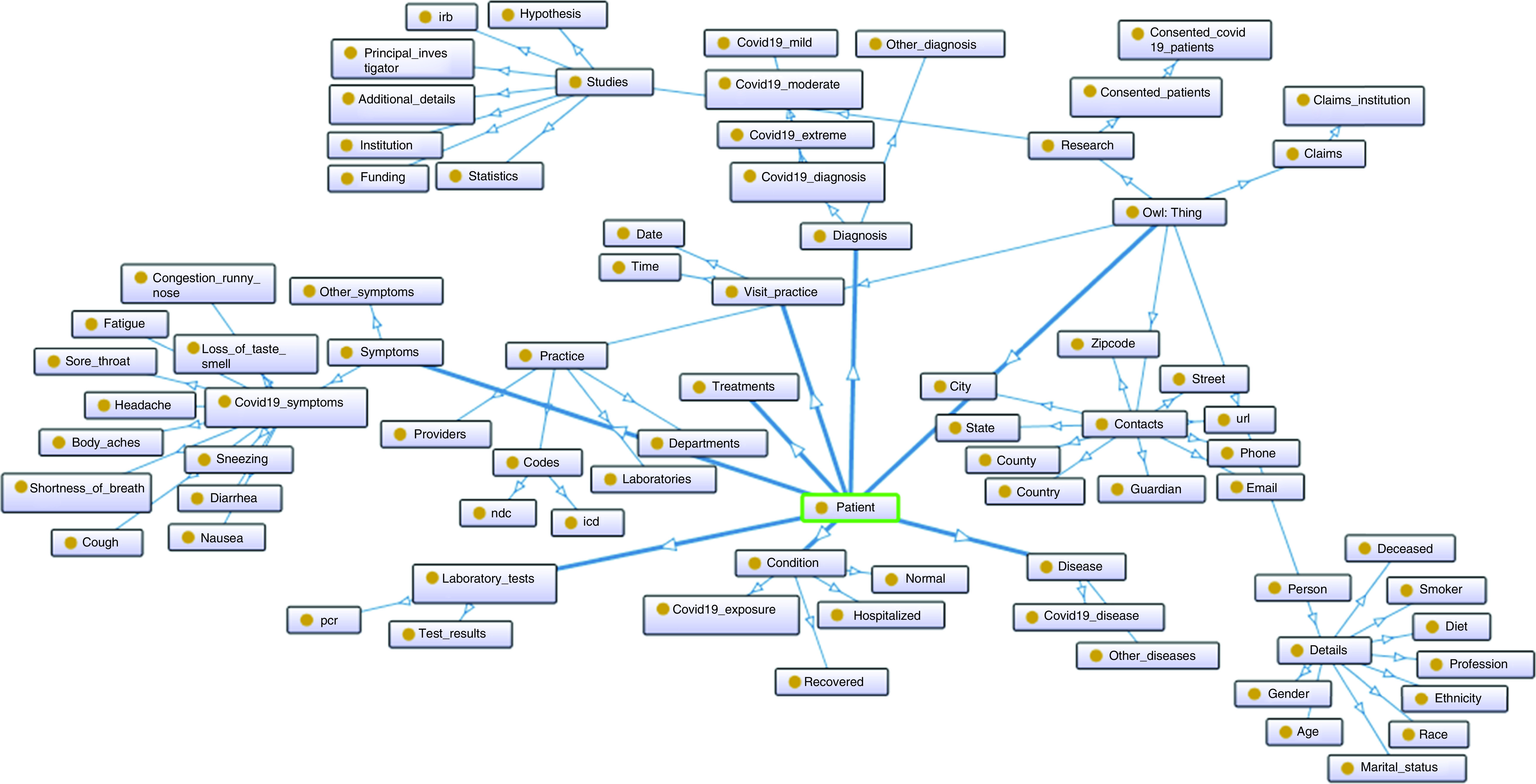
Designed COVID-19 ontology is mainly based on details about persons, patients, symptoms, visits, practices, providers, diagnosis, laboratory tests, disease, treatments, procedures, claims, consent and research.

Our need today is the availability of the open-source healthcare system that can automate repetitive tasks and effectively link data received from different platforms. Furthermore, it can curate a knowledge base of phenotypes and biomarkers of COVID-19 patients to perform longitudinal population studies in order to analyze the effects of treatments and establish relevant scientific studies. As such platforms are not available, we need to develop new ones to help detect and build new predictive models, understand COVID-19 disease mechanisms and identify major causes of morbidity and mortality. Considering the dilemma of establishing whether a patient has COVID-19, when given many numeric input variables that represent different characteristics. We need to collect, generate, analyze and share de-identified healthcare data of the consenting COVID-19 patients to support modeling and training of AI models. Our approach could include the us of logistic regression to weight each input variable with a weighted sum, as a good indicator of the disease. Since health and disease often involve complex interactions, it is important to start analysis by clustering variabilities and commonalities of personalized and population elements. Stratification needs to be based on COVID-19 disease, demographic distribution, clinical features, medications, drugs and biomarkers. Combinations of diagnoses, medications and lab tests will create vast numbers of scenarios that can be efficiently managed and intelligently analyzed by the recommended system, which can help implement personalized treatment and predictive diagnostics of COVID-19 patients. We need implementation of a module, where multiple demographic and phenotypic features will be used to construct cohorts and ontologies from COVID-19 patient and population data (Figure 3). Cohorts and ontology building features must include information based on person (age, deceased, diet, ethnicity, race, gender, marital status, smoker, profession), patient (condition, COVID-19 exposure, hospitalized, normal, recovered), diagnosis (COVID-19 and others), laboratory tests PCR and others, symptoms, treatments, procedures, visits, practice, providers, claims and research.

**Figure 4.**
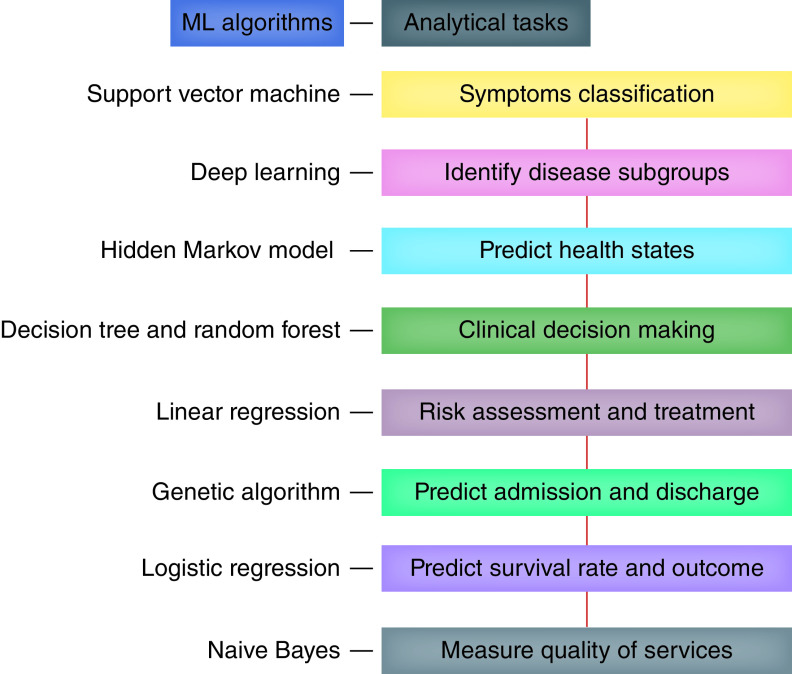
Artificial intelligence nexus: machine learning algorithms and analytical tasks. AI: Artificial intelligence; ML: Machine learning.

We propose the application of the most useful AI and ML algorithms in the healthcare data analytics process to extract features, capture complex non-linear relationships, build up neural networks, cluster groups subjects with similar traits and generate meaningful results from structured clinical data generated from different activities [6]. Here, we suggest a nexus of multiple supervised ML approaches to examine COVID-19 data granularity from population studies to subgroups stratification (Figure 4). This nexus aims to improve the overall provision of the quality healthcare services to the most deserving COVID-19 patients. Nexus starts with the support vector machine [12] for symptoms classification and analysis to improve diagnostic accuracy. Next, apply deep learning [15,16] for the identification of disease subgroups, prognostically relevant risk factors and measuring medication adherence. Hidden Markov Model [17] is linked for predicting COVID-19 patients entering states with a high number of asynchronies, which includes modeling ‘healthy’ and ‘unhealthy’ unobserved health states and mining adverse drug reactions from available healthcare forums for appropriate and alternate therapies. Decision tree and random forest [18] are following to support clinical decision making process by identifying predictors, analyzing data streamed from real-time healthcare monitoring devices (e.g., electrocardiography, pulse oximeter, ventilators) and making mortality predictions. We imply constructing linear regression [19] to ensure treatment appropriateness and risk assessment of COVID-19 by taking mean on decision making and monitoring drug prescribing patterns. At last, we conclude with genetic algorithm [20] to predict scheduling of COVID-19 patient admission, allocation of critical resources and discharge. Additionally, we recommend testing the performance of linear regression [21] for predicting COVID-19 patient survival rates and outcomes of encounters with health providers by identifying discriminatory characteristics and Naive Bayes [22] for measuring the quality of healthcare services.

## Secure clinical & open COVID-19 data management

The significance of heterogeneous healthcare data mining cannot be denied, but the challenges of big data management looms large [23]. The rapid growth in COVID-19 healthcare data sources require robust and automatic translation into improvements for the clinicians and patients at the point of care [1]. Most of the freely available COVID-19 data sources are based on heterogeneous structures. When commercial EHR systems deployed in clinical environments and laboratories are very complicated, rigid and restricted, as those are designed with goals mainly around data entry and retrieval without comprehensive analytics. The greatest challenge here is to deal with the daunting complexity of the COVID-19 data, as complex structures of its databases are based on different number of relations attributed with different numbers of fields, types and noise. Effectively implementing a precision medicine approach for COVID-19 requires a secure information technology framework, preferably integrated into a medical institution [24] and linked to the operational EHR, pathology systems and laboratory information management system. We need to develop secure frameworks to support clinical, phenotypic, epidemiological and computationally analyzed data storage and management of consented COVID-19 patients. Architecture of such system need to meet Health Insurance Portability and Accountability Act, good clinical practice and Institutional Review Board requirements for interfacing clinical databases to streamline data workflow with secured and controlled accessibility and transparency. Contributing globally, we need to contemplate hosting anonymized data to support internal and global research objectives for effectively drawing comparisons with recently generated and earlier existing COVID-19 data sets.

**Figure 5.**
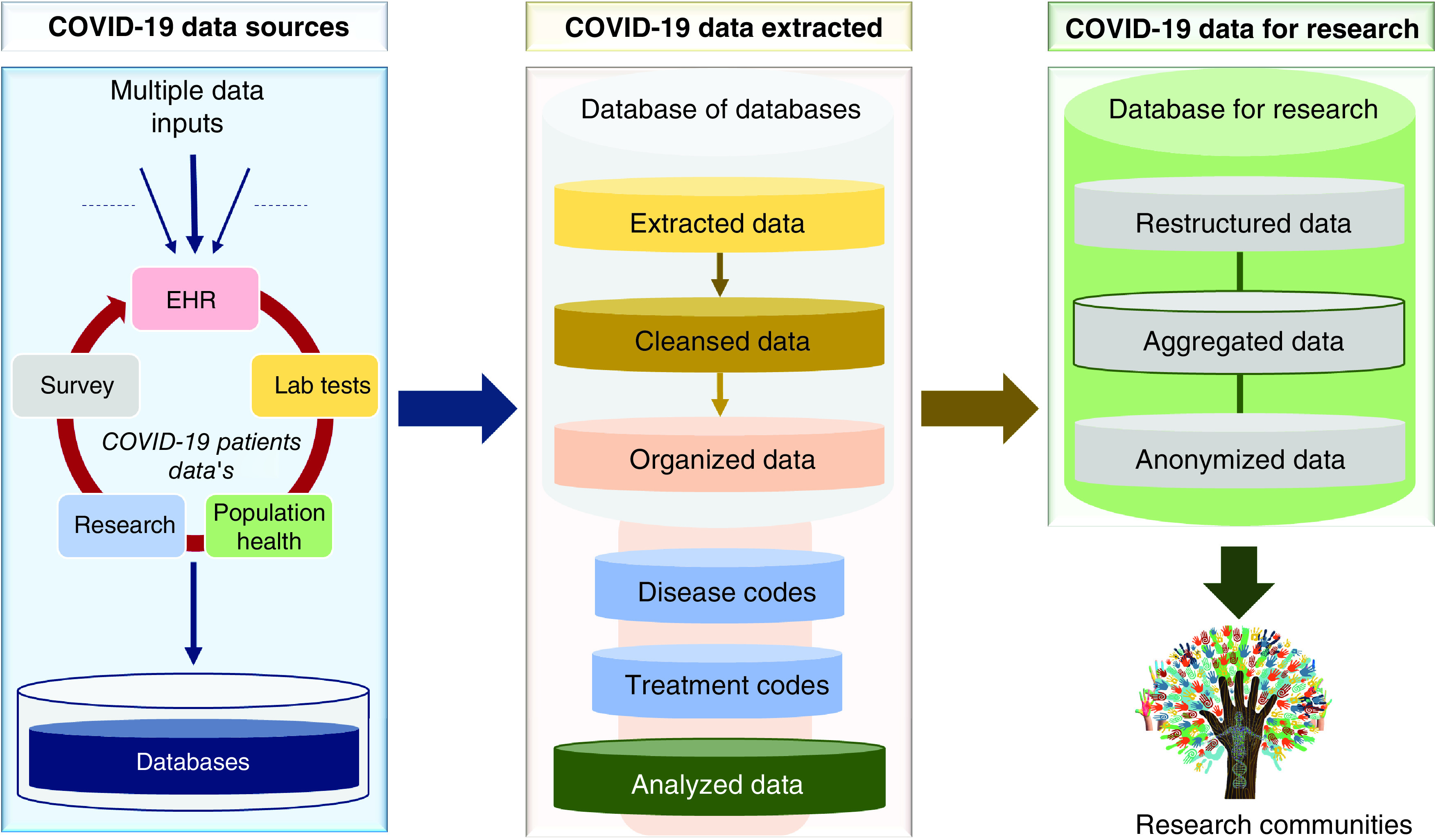
Data flow from multiple data sources to research.

To facilitate efficient and secure COVID-19 data management, we suggest a model of three distributed database servers: data extraction, transfer and load (ETL) from multiple COVID-19 data sources; data cleansing and organization to support COVID-19 data analysis; and data restructure, aggregation and anonymization to publicly share COVID-19 data with research communities (Figure 5). Extended high level of heterogeneity and variety of clinical terminologies have made EHRs and diversified public content processing extremely arduous. Addressing this challenge, we need to develop an intelligent and dynamic data ETL system for efficiently pulling clinical data from different health systems and academic data models [10]. Further extending the scope of existing work, we must develop application programming interface. These will support the overall research community in efficiently ETL and cleansing of multisource COVID-19 data. To invite interested users and data analysts from multidisciplinary backgrounds with little or no programming skills, we should develop user friendly graphical interfaces, which can help in performing complicated biomedical informatics and data science-oriented tasks. Furthermore, this will also equip experienced bioinformatics professionals with more effective methods of data management, integration and analysis.

## Conclusion

The world needs to show solidarity and pull together to identify gaps and challenges in national and international capacities in responding to this global public health crisis. Most of the symptomologies of the COVID-19 are based on common signs and symptom driven diagnosis and treatments. Currently COVID-19 diagnostics are based on clinical assays, when the need of the hour is for deep immune genotyping to generate genomic surveillance data.

## Future perspective

While conducting COVID-19 research studies, it is important to involve diverse and multicultural populations with a wide range of physical, cognitive, mental health and sensory disabilities (e.g., low vision, color blindness, hearing impairment and tremors) and from different age (including adolescents and older adults), gender and racial/ethnic minority groups (e.g., African–Americans, Hispanics/Latinos, American Indians/Alaska Natives, Native Hawaiians and other Pacific Islanders). We recommend addressing ethical, legal and social implications in implementing intelligent health systems for the investigation and consenting of COVID-19 patients, which include maintaining integrity, confidentiality, privacy and security of the consented patients. Incorporating data security standards and best practices will ensure privacy, confidentiality, integrity and security, particularly for healthcare data captured, transferred, analyzed and disseminated. Mobile technology (e.g., iOS and Android-based smart phones and tablets) and consumer wearable devices can be used to for the participant recruitment, consenting and onboarding. Mobile Health Platform [25] can support real-time and automatic digital health data collection, annotation, harmonization, transmission and sharing [26,27]. Furthermore, consumer wearable devices can be helpful in collecting information related to the post-acute Sequelae of COVID-19 (PASC) [28] such as beat-to-beat heart rate, cardiovascular hemodynamics (e.g., systole duration, pulse pressure, pulse volume), respiration rate, skin temperature (temperature, fever detection, calibration for oxygen saturation measurements), cough, anxiety, actigraphy, physical (motion), activity levels and sleep patterns [29,30].

Multidisciplinary teams of investigators need to be assembled including, but not limited to, clinicians, biologist, geneticist, epidemiologists and computer scientists, informaticians and biostatisticians with extensive experience developing customized tools and utilizing real-world data for clinical research [31]. To coordinate data flow, indexing and linkage of all digital health data, we need to develop and use standards-based methodologies to support data interoperability and exchange with the Digital Health Data Repository and data resource core [32]. We recommend using Digital Health Data Repository and data resource core with suitable privacy and security safeguards, for example, recognized encryption protocols and appropriate standards for data harmonization and interoperability. Validated digital health measures need to be implemented for the assessment of the trajectory of acute COVID-19 and PASC. These measures must include: COVID-19 patient’s personal details and medical history; specific symptoms experienced by the COVID-19 patient; geospatial data linked with environmental and social context data (e.g., weather data, socioeconomic deprivation indices); results of COVID-19 testing; duration for being hospitalized; development and resolution; time points and frequency of engagement with COVID-19 patients; and biomarkers (e.g., C-reactive protein, cardiac enzymes and cytokines) [33].

The implementation and growth of AI and ML in healthcare has been incongruent in low- and middle-income countries (LMICs) compared with high-income countries [34]. Major reasons include low budget, lack of computational resources and unavailability of trained personal, specialist medical professionals and investigators for innovative research, development and practice in healthcare. Furthermore, in LMICs, patients have limited access to the high-quality drugs for their personalized treatment (e.g., vaccines for COVID-19), diagnostic imaging hardware for ultrasound and x-ray, surgical infrastructures including operating theaters, medical devices and anesthesia for emergency and pandemic situations and next-generation sequencing facilities for whole-genome (DNA) [35,36], transcriptome (RNA) [37,38] and single-cell sequencing [39] etc. Absence of these healthcare supporting services is causing dearth in the generation of heterogenous clinical and scientific data, which needs to be used in AI and ML-based predictive analysis. To advance the healthcare in LMICs and even in high-income countries, it is required to invest in the resource building for increasing the laboratory capacity and improving data communication methods in the healthcare institutions, including deployment of passable number of qualified clinical workforces and infection preventionists. In future, we need to imply AI and ML-based intelligent and MPH systems, especially in LMICs for dealing with the challenges including, but not limited to, prevention, timely detection and monitoring of infectious, chronic and other complex diseases [40].

**Executive summary**

- The aggregation of artificial intelligence and healthcare data will interlace the threads of the complex landscape of COVID-19 medicine resources, which is currently pervaded by a high level of heterogeneity and lack of standards. Therefore, it is important to address the key issues related to the heterogeneous levels of data granularity, collection, management, privacy and security.
- As traditional data management methods cannot handle the scale and variety of COVID-19 data acquired and generated daily. Meeting essential Health Insurance Portability and Accountability Act, good clinical practice and Institutional Review Board requirements, we postulate the development of a secure and advanced framework for the permanent archiving and sharing of anonymized clinical, phenotypic, epidemiological and computationally analyzed data.
- We propose applying multiple artificial intelligence approaches and effectively utilizing COVID-19 big data to entail all the steps from data collection, storage, processing, mining, analysis and final application. We highlight the need to develop an external cross platform and interactive interface for physician–computer interactions to address the challenges of data integration and clinical exploitation in health systems.
